# Regulation of tartary buckwheat‐resistant starch on intestinal microflora in mice fed with high‐fat diet

**DOI:** 10.1002/fsn3.1601

**Published:** 2020-05-12

**Authors:** Yiming Zhou, Yun Wei, Beibei Yan, Shen Zhao, Xiaoli Zhou

**Affiliations:** ^1^ Department of School of Perfume and Aroma Technology Shanghai Institute of Technology Shanghai China

**Keywords:** intestinal microflora, resistant starch, short‐chain fatty acids, tartary buckwheat

## Abstract

Resistant starch (RS) is closely related to the composition of intestinal flora. Based on many studies on the physiological functions of probiotics and short‐chain fatty acids (SCFAs), it is possible that RS can improve the intestinal health of the host. Therefore, we speculated that tartary buckwheat‐resistant starch (TBRS) can also regulate the intestinal flora disorder caused by high‐fat diet. We randomly divided 36 SPF C57BL/6J mice into low‐fat diet, high‐fat diet (HF‐CS), high‐fat diet supplemented with TBRS (HF‐BRS), and high‐fat diet supplemented with corn‐resistant starch (HF‐CRS). We analyzed the diversity and richness of gut microbiota based on PCR and Illumina high‐throughput sequencing technology. In community abundance, the HF‐BRS group was significantly higher than the other three groups (*p* < .05). TBRS improved the gut microbiota dysbiosis, including decreasing the *Firmicutes*‐to‐*Bacteroidetes* ratios (F/B) and contributing to the growth of *Bacteroides* and *Blautia* as well significantly inhibiting the growth of *Bifidobacterium*, *Faecalibaculum*, and *Erysipelatoclostridium*. We also analyzed the production of SCFAs by GC‐MS, and the concentration of total SCFAs increased in the HF‐CS group. However, TBRS significantly increased the production of SCFAs, especially the propionate concentration compared with the HF‐CRS group (*p* < .05). These results elucidated that TBRS has the potential to improve intestinal health by altering the structure of gut microbiota and increasing the production of SCFAs. Our findings have important implications for TBRS as functional food ingredient to manipulate intestinal microflora.

## Graphical abstract

TBRS has the potential to improve intestinal health by altering the structure of gut microbiota and increasing the production of SCFAs, which was superior to CRS. Our findings have important implications for TBRS as functional food ingredient to manipulate intestinal microflora.

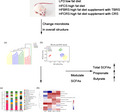

## 1 INTRODUCTION

Tartary buckwheat is a rare crop within the homologous crops of grain and medicine (Aubrecht & Biacs, 2001). It grows mostly in the plateau areas that have poor environments in China. With its high levels of nutritional components (e.g., resistant starch, minerals, antioxidants, flavonoids, high‐quality protein, and dietary fiber; Guo et al., 2012; Liu, Lv, Peng, Shan, & Wang, 2015; Wijngaard & Arendt, 2006) and its ability to prevent and control diseases (Qin, Wang, Shan, Hou, & Ren, 2010), tartary buckwheat has high edible value and high potential in the health food market (Li, Lin, & Corke, 1997). It is noteworthy that the main component of tartary buckwheat is starch, accounting for 70%, and its content of resistant starch (RS) ranges from 13.1% to 22.5% (Liu, Guo, et al., 2016; Liu, Wang, et al., 2016; Liu, Chen, et al., 2016; Zhu, 2016).

RS is resistant to enzymatic hydrolysis in the small intestine and can be fermented by microorganisms in the colon to produce short‐chain fatty acids (SCFAs) that have a variety of physiological effects on the health of the host (Asp & Björck, 1992; Haenen et al., 2013; Hedemann & Knudsen, 2007; Topping & Clifton, 2001). As a constituent of dietary fiber, RS has many physiological functions that are beneficial to human health, such as increasing satiety, reducing the risk of colon cancer and obesity (Kelly et al., 2015), and enhancing mineral absorption (Sajilata, Singhal, & Kulkarni, 2006). Moreover, RS is a nonsticky fiber that does not affect intestinal excretion or nutrient absorption, and its ability to balance the intestinal environment as a beneficial fermentation substrate had been confirmed (Damms‐Machado et al., 2015; He, Sun, Ge, Mu, & Zhu, 2017). For instance, Zhang, Wang, Zheng, Lu, and Zhuang (2013) found that RS could provide Bifidobacterium with nutrients and protect it. According to the literature (Regassa & Nyachoti, 2018), the addition of RS to the diet has the potential to modify the gut microbial community and improve the gut health and function of the host.

A large number of microorganisms coexist with their hosts for a long time and depend on each other to form a highly complex intestinal microecosystem in the intestine of adult animals (Bird, Brown, & Topping, 2000). Intestinal microorganisms not only affect the host's food metabolism and inhibit pathogenic bacteria but also regulate the host's immune response to diseases (Gaweł, Wardas, Niedworok, & Wardas, 2004). Therefore, intestinal microorganisms are particularly important for the physiological and health functions of the host (Shingu, Yoshioka, Nobunaga, & Yoshida, 1985). A previous study revealed that RS can promote the growth and reproduction of *Lactobacillus* and *Bifidobacterium*, thus altering colonic microbial flora (Zeng et al., 2017). Similar results have been reported by Javadi, Shafiei, and Mirzaei (2012), who also observed that the addition of RS increased the relative abundance of *Lactobacillus* significantly, whereas the abundance of harmful bacteria such as *Escherichia coli* and *Bacteroids* decreased significantly. SCFAs produced by RS fermentation by gut microbiota can reduce the intestinal pH value, the growth and reproduction of saprophytes, and the production of carcinogens (Nugent, 2005). In addition, SCFAs have physiological functions of promoting colonic homeostasis (Bhutia & Ganapathy, 2015), regulating immune response, improving intestinal circulation (Smith et al., 2013), and inhibiting the growth of pathogenic microorganisms (Abdul Rahim et al., 2019).

As a dietary intervention, RS that benefits the host by altering intestinal microflora continues to receive widespread attention. At present, most research on tartary buckwheat‐resistant starch (TBRS) focus on its processing characteristics, but little research had involved the study of physiological functions (Gao et al., 2016; Liu, Guo, et al., 2016; Liu, Wang, et al., 2016; Liu, Chen, et al., 2016; Zhou, Zhou, Xiao, Liu, & Chen, 2013). Therefore, it is of great importance to investigate the physiological functions of TBRS. In this study, we used the model of mice fed with a high‐fat diet to evaluate the regulatory effects of TBRS on intestinal flora. We sequenced the microbial DNA extracted from the feces of experimental animals by high‐throughput sequencing technology (Illumina platform). The changes of SCFAs in intestinal contents were detected by gas chromatography–mass spectrometry (GC‐MS) and other detection techniques. The objective of the study was to investigate the regulation of resistant starches in tartary buckwheat on gut microbiota. The results of the study will help to determine the potential use of TBRS as a functional food ingredient and as a raw material selected for food processing.

## 2 MATERIALS AND METHODS

### 2.1 Materials and chemicals

The extraction and determination of composition of TBRS were performed in our previous study (Zhou, Zhao, Jiang, Wei, & Zhou, 2019). All chemicals and reagents were of analytical grade.

### 2.2 Animals and treatment

The experiment was approved by the Animal Care and Use Committee of Shanghai Institute of Technology and was carried out in strict accordance with Chinese animal welfare standards. The experiment used 36 SPF C57BL/6J mice (male; age, 3 weeks; weight, 90–100 g), which were purchased from Shanghai SLAC Laboratory Animal Co., Ltd. We randomly divided all of the mice into the following four treatment groups (*n* = 9), and each group was caged separately: low‐fat diet (LFD), high‐fat diet (HF‐CS), high‐fat diet supplemented with TBRS (HF‐BRS), and high‐fat diet supplemented with corn‐resistant starch (HF‐CRS). Before assignment, a basal diet was given to all mice, which were cohoused for 1 week to acclimate to the laboratory environment. Food and water intake were not restricted during the 12‐week experimental period, and conditions of 25 ± 2°C, 50 ± 10% humidity, and 12‐hr light/dark cycles were maintained throughout the entire study.

### 2.3 Fecal bacteria collection and bacterial genomic DNA extraction

Fresh fecal samples were separately collected from the colon during the final 5 days, frozen in liquid nitrogen, and stored at −80°C for future DNA extraction and molecular microbiological analysis. Gut microbiota DNA was extracted from the samples according to the manufacturer's instructions of the genomic DNA extraction kit (ComWin Biotech Co. Ltd.).

### 2.4 Polymerase chain reaction amplification and sequencing

We checked the extracted genomic DNA by agarose gel electrophoresis and amplified the V3–V4 region of the bacterial 16S ribosomal RNA gene by polymerase chain reaction (PCR) using synthetic primers with a specific barcode. We performed the analysis of the mixed PCR product of the same sample by 2% agarose gel electrophoresis. We used an AxyPrep DNA Gel Extraction Kit (Axygen Biosciences) to extract the amplicons from 2% agarose gel and to purify them according to the manufacturer's guidelines. In addition, we used the QuantiFluor‐ST (Promega) for amplicon quantification and conducted a corresponding proportion of mixing according to the requirements for the sequencing quantity of each sample (Zhu, Ma, Ding, Jiang, & Fang, 2018).

After PCR amplification, we aggregated the purified amplicons by an Illumina Sequencer MiSeq platform by Majorbio Bio‐Pharm Technology Co. Ltd. following the standard protocols and constructed the amplicon libraries for high‐throughput sequencing (Liu, Guo, et al., 2016; Liu, Wang, et al., 2016; Liu, Chen, et al., 2016).

### 2.5 Sequencing data processing

After splicing and filtering the Illumina paired‐end reads, we clustered all valid sequencing reads into operational taxonomic units (OTUs) based on 97% pairwise identity against the reference database using UCLUST software, discarding reads that failed to match the reference sequences (Dong, Song, Wang, Mu, & Li, 2017). We used the number of OTUs as a measure of microbiome richness and used the standard sequence number corresponding to the sample with the least sequences to normalize the OTUs abundance information. Subsequently, we conducted all alpha and beta diversity analyses according to these normalized output data. For alpha diversity analysis, we obtained curves of rarefaction and Shannon by rarifying the OTUs and calculated the indexes of Ace, Chao1, and Shannon curves to assess community richness and diversity. We used beta diversity analysis to evaluate differences among the samples in each species complexity, including hierarchal cluster analysis, heat‐map cluster analysis, and principal coordinate analysis (PCoA).

### 2.6 Determination of SCFAs in feces

We determined the SCFAs in the frozen feces samples using the following modified methods: We put 1 g of lyophilized stool samples into a centrifugal tube and gently suspended the sample in 1.0 ml of ultrapure water. The sample was vortex mixed for 30 s and then centrifuged at 15,000 *g* for 15 min at room temperature. We added 25% metaphosphate in the ratio of v:v = 9:1 to the collected supernatant for 3 hr, using a GCMS‐QP2010 Ultra (Shimadzu) for GC‐MS analysis.

### 2.7 Statistical analysis

We analyzed data using one‐way analysis of variance (ANOVA) tests to determine the effect of TBRS on the microbial population. All of the values in the tables and figures are mean ± standard error of the means. We performed statistical analysis using SPSS 19.0. A *p*‐value of less than .05 was considered to be statistically significant.

## 3 RESULTS

### 3.1 General OTUs distribution statistics information

**Figure 1:**
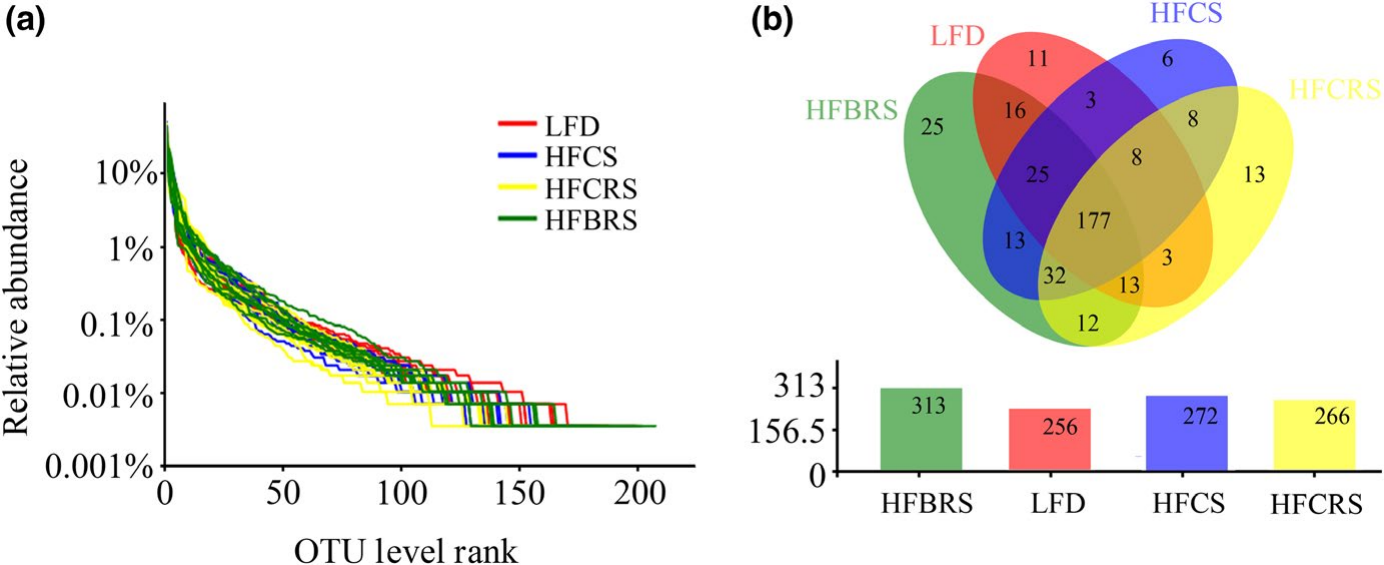
(a) OTU rank curves of gut microbiota of each sample. (b) Core bacterial OTUs in mice from different treatment groups

We obtained a total of 1,812,241 high‐quality sequences of the V3–V4 region of the 16S rRNA from the 36 fecal samples. The average sequence number was 50,340, with the maximum being 79,624 and the minimum being 33,552, and the average length was 438 bp. All of the sequences were clustered into 1,107 OTUs at a 97% similarity level, and the HF‐BRS group was 313, which was the most OTUs in the four groups. Every line in the rarefaction curves represented the OTU distribution of each sample; the gentler the curve, the more uniform the OTU distribution. This result revealed that OTUs were evenly distributed throughout the samples (Figure 1a). The taxon abundance of each sample was generated into 15 phyla, 23 classes, 37 orders, 59 families, 134 genera, and 215 species. We created a Venn diagram to summarize the number of common OTUs assigned at a 97% sequence similarity threshold among the four groups. We determined the core community on the basis of the 365 OTUs detected in every fecal sample. We identified about 177 OTUs as the core bacterial OTUs accounting for 48.49% of the total OUTs. The number of OTUs in each group was as follows: 11 (3.01%) for the LFD group, 6 (1.64%) for the HFCS group, 25 (68.50%) for the HF‐BRS group, and 13 (3.56%) for the HF‐CRS group (Figure 1b).

### 3.2 Alpha diversity analysis of gut microbiota in the mice

**Figure 2:**
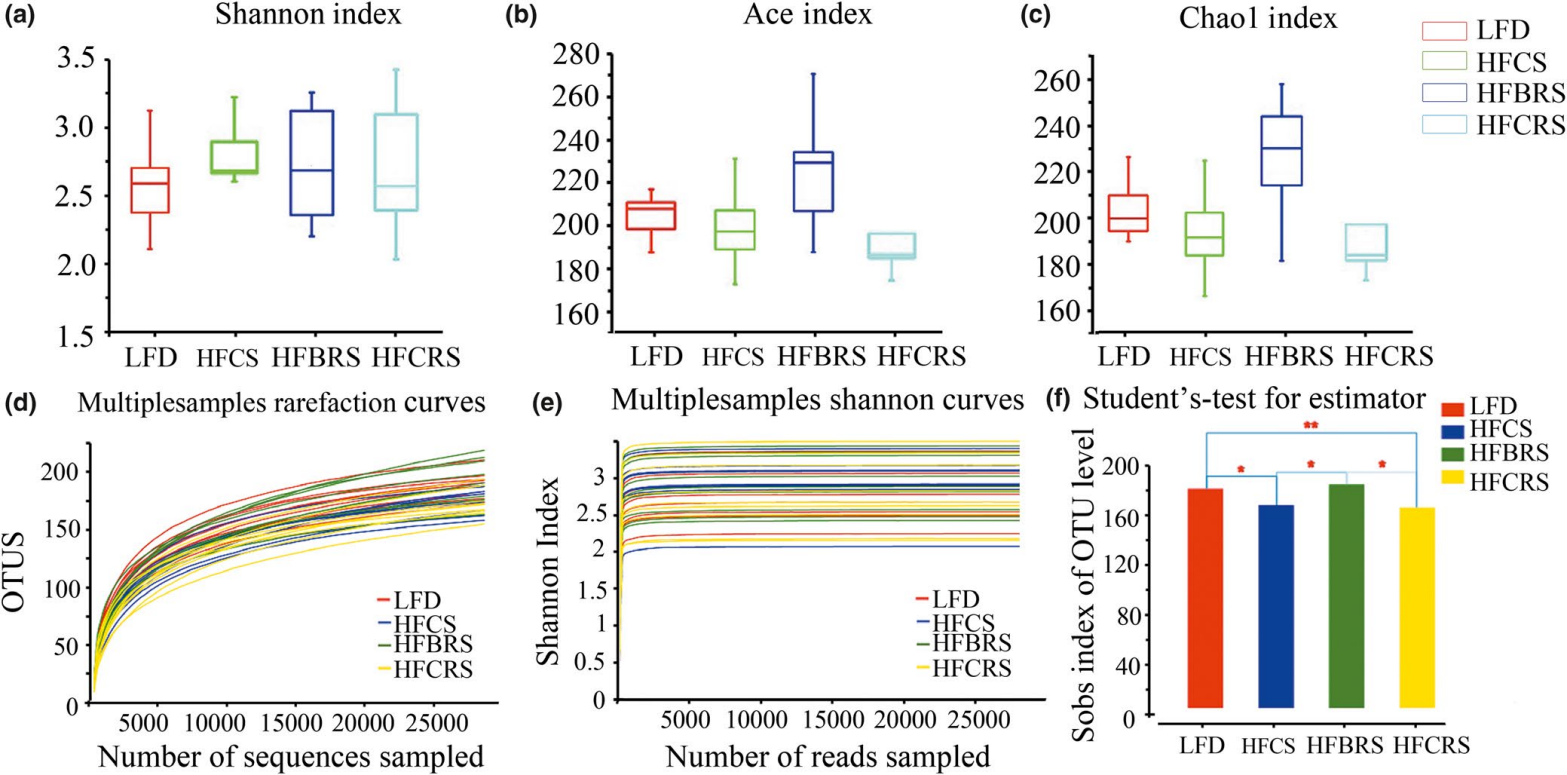
Responses of the diversity and richness of the gut microbiota in four groups. The Shannon index (a), Ace index (b), and Chao1 index (c) of each group. Values are presented as mean ± *SD* from experiments in triplicate. Differences were assessed by one‐way ANOVA followed by the LSD post hoc test. Different letters represent significant differences *p* < .05. Rarefaction curves (d) and Shannon curves (e) of gut microbiota for each sample. (f) The histogram of intergroup difference test among the four groups (.01 < *p* ≤ .05 marked as *, .001 < *p* ≤ .01 marked as **)

Alpha diversity was related to two main factors: species richness and species evenness in individual distribution. These two factors have been used to describe the relative richness or proportion of individuals in a species (Ventura et al., 2009). We investigated alterations of community diversity index (expressed as Shannon) and community abundance (expressed as ACE and Chao1) in response to different diet treatments. There was no significant difference in the Shannon index among the four groups (*p* < .05; Figure 2a). We observed lower Ace and Chao1 indexes in the HF‐CS group, however, as opposed to the LFD group (Figure 2b,c), which suggested that a high‐fat diet decreased the richness of the bacterial community. After intervention of CRS, the Ace and Chao1 values did not change significantly (*p* < .05). A high‐fat diet cosupplemented with TBRS, however, resulted in higher community richness than that of the HF‐CS group, which was closer to the LFD group. Additionally, as a measure of sampling completeness, Good's coverage ranged to 99%.

The curves of rarefaction on OTU level and Shannon reached a plateau phase (Figure 2d,e), indicating that the amount of sequencing data was reasonable and sufficiently large to reflect the vast majority of microbial diversity information in the sample and demonstrating that most bacterial species in all samples had been captured (Amato et al., 2013). Additionally, we calculated intergroup differences and found that there was no significant difference between the HF‐BRS group and the LFD group, whereas there was a highly significant difference between the HF‐CRS group and the LFD group (*p* < .05; Figure 2f).

### 3.3 Beta diversity analysis of gut microbiota in the mice

**Figure 3:**
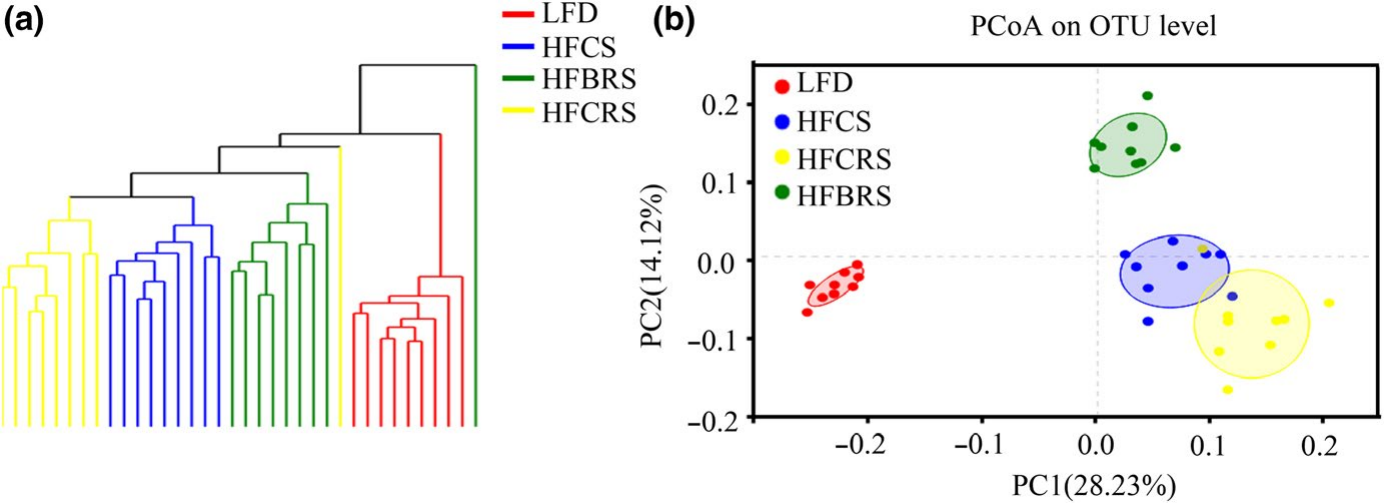
Beta diversity analysis of four groups at the OTU level. (a) Hierarchically clustering tree on the OTU level. (b) PCoA on OUT level

The unweighted UniFrac analysis indicated that hierarchical clustering and PCoA could be used to analyze similarities and differences in the community composition of all samples (Wang et al., 2017). A hierarchical clustering tree on the OTU level of the different samples showed that the difference between the HF‐BRS group and the LFD group was smaller than that between the HFCS group and the LFD group (Figure 3a). A clear separation was evident between the LFD group and the other three high‐fat diet samples. All four groups presented a distinct clustering of microbiota composition, but there was partial overlap between the HF‐CS and HF‐CRS groups. Furthermore, the HF‐BRS group had a better similar structure to the LFD group than that of the HF‐CRS group (Figure 3b).

### 3.4 Distribution of the fecal microbiota based on phylum level

**Figure 4:**
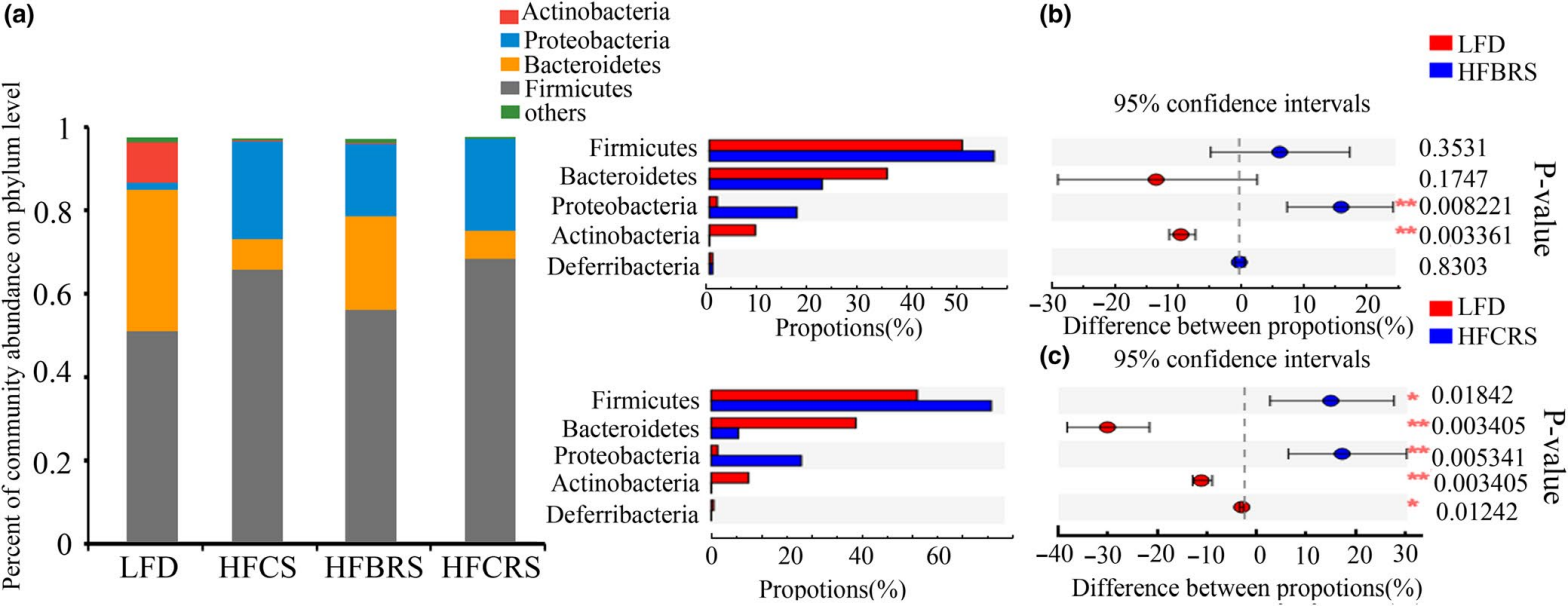
Bacterial community abundance at the phylum level of each group. (a) Bar chart of taxonomic distribution at the phylum level. Different color bars represent different bacterial phyla. (b) and (c) The Wilcoxon rank‐sum test bar plot of mice between groups at the phylum level (.01 < *p* ≤ .05 marked as *, .001 < *p* ≤ .01 marked as **)

As shown in Figure 4a, the top four phyla were *Firmicutes*, *Bacteroidetes*, *Proteobacteria*, and *Actinobacteria*. The relative abundance of *Firmicutes* and *Proteobacteria* increased significantly, and the relative abundance of *Bacteroidetes* and *Actinobacteria* decreased significantly in the HF‐CS group, although these levels were restored after TBRS treatment. Consistent with the phylum‐level analysis, there was no significant difference in the relative abundance of *Firmicutes* and *Bacteroidetes* between the LFD and HF‐BRS groups (Figure 4b). There was, however, a significant difference in the relative abundance of *Firmicutes* and *Bacteroidetes* after CRS intervention (Figure 4c). The relative abundance of *Actinobacteria* and *Proteobacteria* had a significant difference in both the HF‐BRS group and the HF‐CRS group (*p* < .05). Moreover, the treatment with TBRS reduced the F/B, which was closer to that of the LFD group (*p* < .05; Table 1). These results demonstrated that TBRS was superior to CRS in improving the structure disorder of gut microbiota in mice fed a high‐fat diet.

**Table 1:** The ratio of *Firmicutes* and *Bacteroides* in the intestinal tract between groups

Group	LFD	HF‐CS	HF‐BRS	HF‐CRS
F/B	1.44 ± 0.88^a^	8.75 ± 0.81^b^	2.52 ± 0.54^a^	8.5 ± 0.79^b^

Notes

Values were expressed as the mean ± *SD* from experiments in triplicate (*n* = 9). The data were analyzed by one‐way ANOVA followed by the LSD post hoc test.

Abbreviations: HF‐BRS: high‐fat diet supplemented with tartary buckwheat‐resistant starch; HF‐CRS: high‐fat diet supplemented with corn‐resistant starch; HF‐CS: high‐fat diet; LFD: low‐fat diet.

Different letters represent significant differences *p* < .05.

### 3.5 Distribution of the fecal microbiota based on genus level

**Figure 5:**
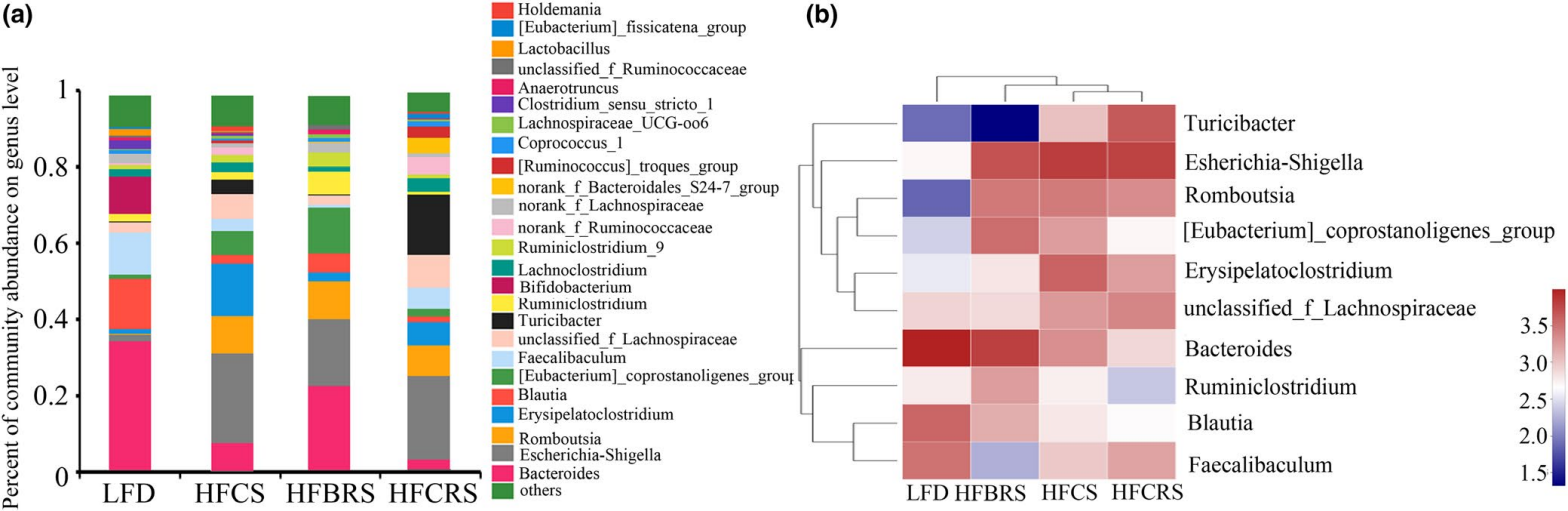
Bacterial community abundance at genus level of each group. (a) Bar chart of taxonomic distribution at the genus level. Different color bars represent different bacterial genera. (b) Heat map of the 10 genera with the highest frequency and relative abundance

We performed a genus‐level analysis to further exhibit differences among the four groups, showing results similar to those observed at the phylum level. The microbial community structure of the intestinal tract of each group had a high diversity at the genus level in more than 20 species. The dominant genus was *Bacteroides*, *Escherichia‐Shigella*, *Romboutsia*, *Erysipelatoclostridium*, and *Blautia*. TBRS supplementation significantly decreased the relative abundance of *Erysipelatoclostridium* belonging to the *Firmicutes* phylum and *Escherichia‐Shigella*, which were harmful to human health in the HF‐CS group, and increased the relative abundance of *Bacteroides* and *Blautia*. Notably, the relative abundance of *Bifidobacterium* and *Faecalibaculum* was not restored after being reduced by a high‐fat diet, and the relative abundance of *Turicibacter* increased significantly after the CRS treatment (Figure 5a). It was evident that there were differences between the LFD group and the other three high‐fat diet groups. The HF‐BRS group and the LFD group, however, had the most similar community compositions (Figure 5b).

### 3.6 Total content of SCFAs in mice feces

As shown in Table 2, HF‐CS feeding significantly increased the concentration of acetate and propionate, as well as the levels of total SCFAs, but there was no significant difference in the concentration of butyrate (*p* < .05). After the two RS interventions, the levels of total SCFAs increased significantly, despite the fact that there was no statistical difference between the HF‐BRS group and the HF‐CRS group (*p* < .05). More concretely, CRS increased the concentration only of acetate, but TBRS increased the concentration of propionate and butyrate.

**Table 2:** Concentration of SCFAs in samples from different treatment groups

Group	LFD	HF‐CS	HF‐BRS	HF‐CRS
Acetate (μmol/g)	20.77 ± 1.04^a^	31.36 ± 2.12^b^	30.00 ± 1.00^b^	33.78 ± 3.04^c^
Propionate (μmol/g)	8.95 ± 0.58^a^	11.55 ± 2.62^c^	20.27 ± 0.90^d^	16.60 ± 2.19^c^
Butyrate (μmol/g)	8.43 ± 0.72^a^	8.77 ± 1.49^a^	10.00 ± 0.83^b^	8.95 ± 0.57^a^
Total acid (μmol/g)	41.34 ± 3.01^a^	54.72 ± 6.24^b^	62.57 ± 2.53^c^	61.59 ± 5.05^c^

Notes

Values were expressed as the mean ± *SD* from experiments in triplicate (*n* = 9). The data were analyzed by one‐way ANOVA followed by the LSD post hoc test.

Abbreviations: HF‐BRS: high‐fat diet supplemented with tartary buckwheat‐resistant starch; HF‐CRS: high‐fat diet supplemented with corn‐resistant starch; HF‐CS: high‐fat diet; LFD: low‐fat diet; SCFAs: short‐chain fatty acids.

Different letters represent significant differences *p* < .05.

## 4 DISCUSSION

Modern environments, especially diet, have a tremendous impact on the formation of our microbial communities, which has resulted in an increased risk for metabolic syndrome and other common diseases. Gut microbes with special composition in the intestine may be the main regulators of host metabolism, which promotes the interaction between functional food and host health (Turnbaugh et al., 2006). An association between gut microbiota dysbiosis and a high‐fat diet, which include a reduction in bacterial richness and diversity, has been confirmed profoundly by several studies. Dietary intervention affects the composition of gut microbiota, as well as the unhealthy conditions caused by microbial imbalance (Rogers & Aronoff, 2015). In this study, we used a high‐throughput Miseq sequencing technique to observe the alternation in bacterial richness and diversity in mice fed a high‐fat diet. The results suggested that a high‐fat diet induced the gut microbiota dysbiosis, which coincided with the findings of Cani et al. (2007). TBRS intake resulted in significant shifts in the overall intestinal microflora structure, partially improving the structural dysbiosis induced by the high‐fat diet.

Few studies have investigated the effect of dietary TBRS on complex gut microbiota. As the dominant bacterial communities in the intestine, alternations of *Firmicutes* and *Bacteroidetes* in response to various diets have been observed. Furthermore, *Firmicutes* decompose saturated fatty acids in the intestine effectively. *Bacteroides* are a group of bacteria that function as carbohydrate fermentation and also affect bile acid and steroid metabolism (Hold, Pryde, Russell, Elizabeth, & Flint, 2002). As the main provider of bile hydrolase gene, *Bacteroides* increased can enhance the activity of bile hydrolase, thus reduce the abundance of 7‐α/β steroid dehydrogenation rate limiting enzyme which encodes the production of secondary bile acid (Gu et al., 2017). In our study, a high‐fat diet decreased the proportion of *Bacteroidetes* and *Actinobacteria* and increased the proportion of *Firmicutes* and *Proteobacteria*, which was consistent with a previous study (Yu, Guo, Shen, & Shan, 2016). After TBRS intervention, there was an increase in the abundance of *Bacteroidetes* and a decrease in *Firmicutes*. This result might have been related to the production of SCFA contributing to the growth of certain microbiota in the colon. In addition, *Firmicutes* may be weakly tolerant to an acidic environment and could be attributed to the increased production of SCFA. Previous studies have shown that high‐fat‐diet‐induced obese mice had lower F/B associated with the energy metabolism of the host than control mice (Qiao et al., 2014). We obtained the opposite results: the average F/B in the LFD, HF‐CS, HF‐BRS, and HF‐CRS groups was 1.44, 8.75, 2.52, and 8.5, respectively, and the increased F/B was almost restored upon TBRS intake. In addition, HF‐CS feeding decreased the proportion of beneficial bacteria, such as Bifidobacterium and *Bacteroides*, directly applied to induce intestinal immunity, and improve the host immune system (Yuan, Shi, Meng, & Wang, 2018). In contrast, a TBRS‐rich diet increased the relative abundance of *Bacteroides* and *Blautia*, both of which could produce SCFAs, and decreased the proportion of pathogenic bacteria, including *Erysipelatoclostridium* and *Escherichia‐Shigella*. These results indicated that TBRS could alleviate the intestinal microflora disorder caused by a high‐fat diet.

SCFAs, which are products of gut bacteria fermentation of undigested carbohydrates, mainly include acetate, propionate, and butyrate, of which butyrate is well known to be the most beneficial to intestinal health (Asarat, Vasiljevic, Apostolopoulos, & Donkor, 2015). Therefore, the relative concentration of the major SCFAs altered by gut microbiota has the potential to generate important physiological consequences. Both TBRS and CRS increased the concentrations of SCFAs in the colons of mice. Some differences existed between the two RS, which may have been related to alternations in the abundance of SCFA‐producing bacteria in the intestinal microflora that resulted from these RS. For instance, an increase in the relative abundance of dominant propionate‐producing *Bacteroidetes* in the TBRS‐treated group may have contributed to an increased propionate concentration (Flint, Scott, Louis, & Duncan, 2012). The relative abundance of acetate‐producing *Blautia* was higher in the HF‐BRS group than in the HF‐CRS group, but the concentration of acetate was lower than that in the HF‐CRS group, which may have been related to no longer using *Bacteroides* with acetate as the metabolic substrate. Furthermore, the relative abundance of butyrate‐producing *Firmicutes* did not change significantly in either of the two RS intervention groups, but a discrepancy existed in the butyrate concentration. This might have been related to the higher digestion of saccharides in TBRS, and additional experiments are essential to explain these results.

## 5 CONCLUSION

Altered gut microbiota that resulted from TBRS intervention provided the theoretical evidence necessary for further understanding the use of TBRS as a functional food. Our study explored the alteration of SCFAs produced by intestinal microflora fermentation, but the mechanism of how TBRS influenced the gut microbiota and SCFAs requires further studies for verification of these results.

## CONFLICT OF INTEREST

The authors declare that they have no conflicts of interest.

## ETHICAL APPROVAL

All procedures were in strict accordance with Chinese animal welfare standards and were approved by the Animal Care and Use Committee of Shanghai Institute of Technology.

## Informed consent

Written informed consent was obtained from all study participants.
